# What we do and do not know about women and kidney diseases; questions unanswered and answers unquestioned: reflection on World Kidney Day and International Woman’s Day

**DOI:** 10.1186/s12882-018-0864-y

**Published:** 2018-03-15

**Authors:** Giorgina B. Piccoli, Mona Alrukhaimi, Zhi-Hong Liu, Elena Zakharova, Adeera Levin, On behalf of the World Kidney Day Steering Committee, Philip Kam Tao Li, Guillermo Garcia-Garcia, Mohammed Benghanem-Gharbi, Kamyar Kalantar-Zadeh, Charles Kernahan, Latha Kumaraswami, Giorgina Barbara Piccoli, Gamal Saadi, Louise Fox, Elena Zakharova, Sharon Andreoli

**Affiliations:** 1 Department of Clinical and Biological Sciences, University of Torino, Torino, Italy; 2 Nephrology, Centre Hospitalier Le Mans, Le Mans, France; 3 Department of Medicine, Dubai Medical College, Dubai, United Arab Emirates; 4 National Clinical Research Center of Kidney Diseases, Jinling Hospital, Nanjing University School of Medicine, Nanjing, China; 5 Nephrology, Moscow City Hospital n.a. S.P. Botkin, Moscow, Russian Federation; 6 Nephrology, Moscow State University of Medicine and Dentistry, Moscow, Russian Federation; 7 Nephrology, Russian Medical Academy of Continuous Professional Education, Moscow, Russian Federation; 8 Department of Medicine, Division of Nephrology, University of British Columbia, Vancouver, BC Canada

**Keywords:** Women, Access to care, Kidney health, Acute and chronic kidney disease, Inequities

## Abstract

Chronic Kidney Disease affects approximately 10% of the world’s adult population: it is within the top 20 causes of death worldwide, and its impact on patients and their families can be devastating. World Kidney Day and International Women’s Day in 2018 coincide, thus offering an opportunity to reflect on the importance of women’s health and specifically their kidney health, on the community, and the next generations, as well as to strive to be more curious about the unique aspects of kidney disease in women so that we may apply those learnings more broadly.

Girls and women, who make up approximately 50% of the world’s population, are important contributors to society and their families. Gender differences continue to exist around the world in access to education, medical care, and participation in clinical studies. Pregnancy is a unique state for women, offering an opportunity for diagnosis of kidney disease, but also a state where acute and chronic kidney diseases may manifest, and which may impact future generations with respect to kidney health. There are various autoimmune and other conditions that are more likely to impact women with profound consequences for child bearing, and on the fetus. Women have different complications on dialysis than men, and are more likely to be donors than recipients of kidney transplants.

In this editorial, we focus on what we do and do not know about women, kidney health, and kidney disease, and what we might learn in the future to improve outcomes worldwide.

## Introduction

Chronic Kidney Disease (CKD) affects approximately 10% of the world’s adult population: it is within the top 20 causes of death worldwide [1], and its impact on patients and their families can be devastating. World Kidney Day and International Women’s Day in 2018 coincide, thus offering an opportunity to reflect on the importance of women’ s health and specifically their kidney health, on the community, and the next generations; as well as to strive to be more curious about the unique aspects of kidney disease in women, so that we may apply those learnings more broadly.

**Fig. 1:**
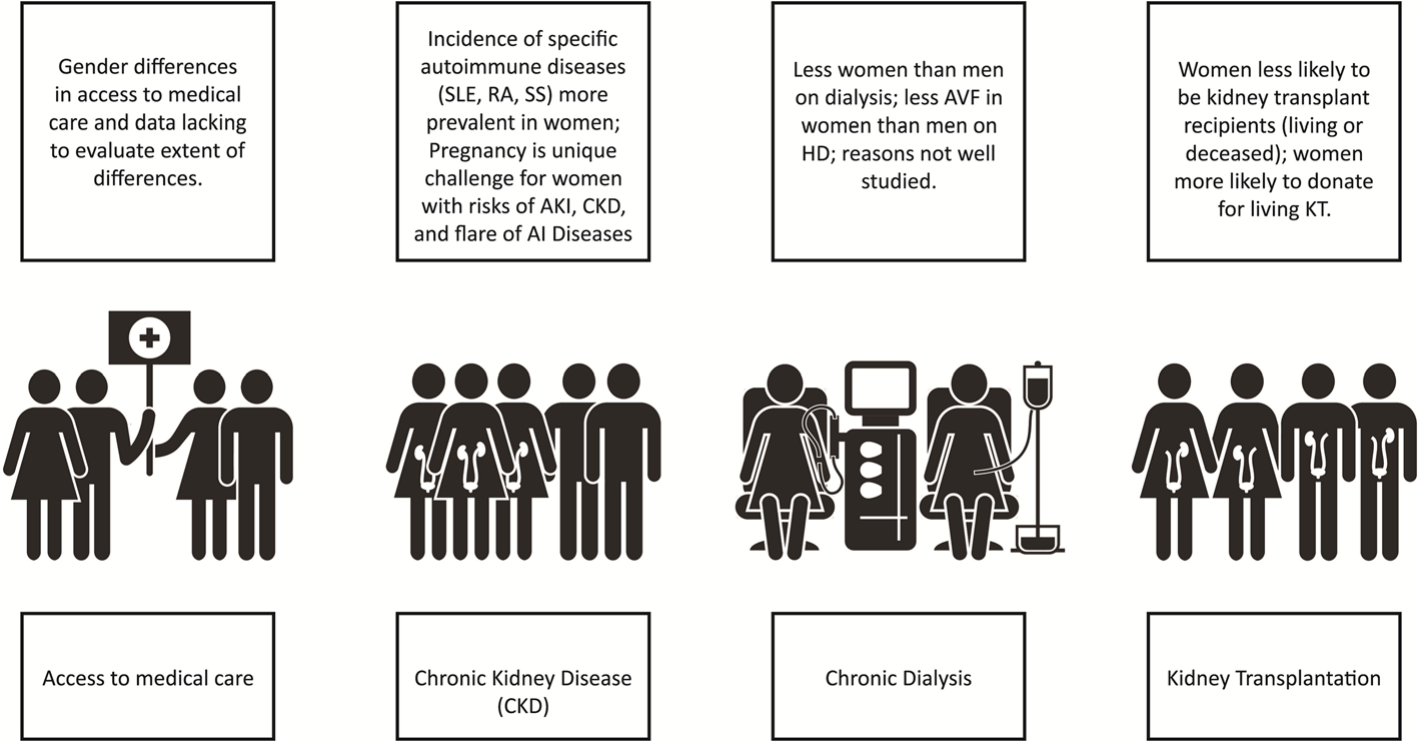
Sex differences throughout the continuum of CKD care. SLE = Systemic Lupus Erythematosus; RA = Rheumatoid Arthritis; SS = Systemic Scleroderma; AKI = acute kidney injury; CKD = chronic kidney disease; AI = autoimmune; AVF = arteriovenous fistula; HD = hemodialysis; KT = kidney transplant

Girls and women, who make up approximately 50% of the world’s population, are important contributors to society and their families. Besides childbearing, women are essential in childrearing and contribute to sustaining family and community health. Women in the 21^st^ century continue to strive for equity in business, commerce, and professional endeavours, while recognizing that in many situations, equity does not exist. In various locations around the world, access to education and medical care is not equitable amongst men and women; women remain under-represented in many clinical research studies, thus limiting the evidence base on which to make recommendations to ensure best outcomes (Fig. 1).

In this editorial, we focus on what we do and do not know about women’s kidney health and kidney disease, and what we might learn in the future to improve outcomes for all.

## Kidney Health and Women’s Health: a case for optimizing outcomes for present and future generations

### What we know and do not know

Pregnancy is a unique challenge and is a major cause of acute kidney injury (AKI) in women of childbearing age; AKI and pre-eclampsia (PE) may lead to subsequent CKD, but the entity of the risk is not completely known [2–5]. CKD has a negative effect on pregnancy even at very early stages [6, 7]. The risks increase with CKD progression thus posing potentially challenging ethical issues around conception and maintaining of pregnancies [6–8]. We do know that PE increases the probability of hypertension and CKD in later years, but we have not evaluated a surveillance or reno-protective strategy to determine if progressive loss of kidney function can be attenuated [9–12].

Specific systemic conditions like Systemic Lupus Erythematosus (SLE), Rheumatoid Arthritis (RA), and Systemic Scleroderma (SS), are more likely to affect women than men. We do not know the relative contribution of these acute and chronic conditions on progression to end-stage renal disease (ESRD) in women.

In CKD cohorts, the prevalence in women is always less than in men, and they have slower progression to ESRD [13–15]. We do not know why and how much of this is due to differences in identification of kidney impairment, different access to care, or true difference in disease severity and prevalence.

Women with CKD have a higher cardiovascular risk than women without CKD [16]; but their risk is still lower than that of men with similar degrees of kidney impairment. In hemodialysis cohorts, there are differences in vascular access types in women versus men, which may be due to biological or systemic factors. In some locations there is differential use of peritoneal and hemodialysis in women and men.

Women are more likely to donate kidneys for transplantation than to receive them. We do not know if this is because of the differential incidence of CKD in men vs women, cultural factors, or other reasons.

There remain gender differences in access to care in different regions of the world, and we do not have data to directly evaluate the extent of these differences, in the poorest parts of the world in particular.

## Pregnancy, preeclampsia, pregnancy-induced hypertensive disorders, and fetal health. The importance of women’s health to present and future kidney health

### What we know

PE is the principal cause of AKI and maternal death, particularly in developing countries [2, 17]. Pregnancy is the most common cause of AKI in women of childbearing age [10, 18, 19]. Several diseases and conditions, besides PE, hypertensive disorders of pregnancy, and CKD, can lead to pregnancy-related AKI. Causes vary in different regions. Septic abortion after an illegal procedure is the leading cause of early AKI in countries where legal abortions are not available, while PE after assisted fertilization is becoming a leading cause in developed countries [12, 20–22].

PE and hypertensive disorders of pregnancy occur in 3-10% of all pregnancies [2, 3, 18]; in these disorders the kidney is the main target of an unbalanced pro-angiogenic and anti-angiogenic derangement, leading to hypertension, proteinuria, and widespread endothelial damage. The incidence of PE, higher in low-middle income countries (possibly reflecting undiagnosed predisposing diseases), peaks at the extremes of reproductive age for reasons mentioned above [12, 20–22].

**Fig. 2:**
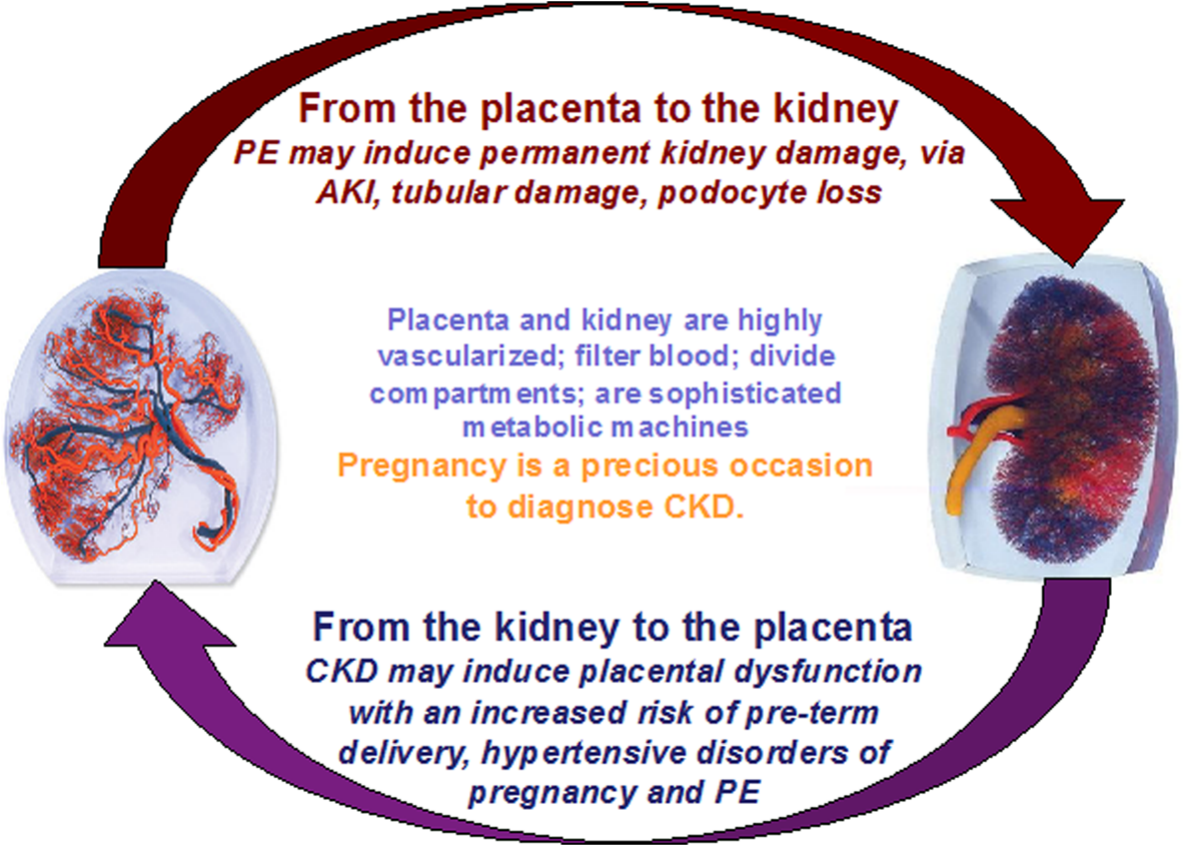
Pregnancy and kidney function: complex interactions between 2 organs, the kidney and placenta. PE = preeclampsia; AKI = acute kidney injury; CKD = chronic kidney disease

The relationship between kidney and placenta is biunivocal, and the presence of CKD is a risk factor for PE and hypertensive disorders of pregnancy (Fig. 2). Besides CKD, other conditions cited as risk factors for PE (diabetes, immunologic diseases, baseline hypertension, obesity, and metabolic syndrome), are also risk factors for CKD. Given that even minor alterations of kidney function are present in many of these disorders, the importance of kidney function is indirectly recognized in the development of PE. Newer definitions of PE recognize differences between “placental” and “maternal” causes of PE, based on novel angiogenic-antiangiogenic markers [23, 24], which may be important for management during and after pregnancy.

*There are long term effects of PE on both maternal and fetal health, but this remains an area of active research with many unknowns.*

PE is a risk factor for the future development of CKD and ESRD in the mother [3–5]. The reasons are not fully understood; podocyte loss is a hallmark of PE, suggesting permanent glomerular damage [25]. Endotheliosis, associated with PE, but also found in normal pregnancies, may herald glomerulosclerosis; tubular and vascular damage may co-exist [26, 27].

Besides maternal risks, PE is associated with intrauterine and perinatal death, preterm delivery, and restricted intrauterine growth; the latter two are linked to “small babies” [2, 3, 5]. Small babies and preterm babies have highly increased risks of neurological deficits and postnatal complications, especially sepsis [28–32]. The risks may be higher in low-income countries, since survival and deficit-free survival depend on the provision of postnatal intensive care [20, 21]. In the long term, small babies are at risk for the development of diabetes, metabolic syndrome, cardiovascular diseases (CVDs), and CKD in adulthood [33–37]. Since kidney development is completed in the last phases of pregnancy, delayed, insufficient kidney growth, resulting in low nephron number is probably the basis of the increased risk of CKD and hypertension in small for gestational age, and preterm babies [33–37].

## Pregnancy in chronic kidney disease, dialysis, and transplantation

### What we know

#### Chronic kidney disease

CKD is a risk factor for adverse pregnancy outcomes from its early stages (Table 1) [6, 38, 39]. The risks increase from CKD stage 1 to CKD stage 5, and may be higher in glomerular nephropathies, autoimmune diseases, and diabetic nephropathy [6, 7, 38–41]. Results of pregnancy after kidney donation suggest that reduction of kidney parenchyma may be associated with a higher risk of PE and hypertensive disorders of pregnancy [42, 43].

**Table 1:** Adverse pregnancy outcomes in patients with chronic kidney disease and in their offspring

Term	Definition	Main Issues
Maternal death	Death in pregnancy or within 1 week-1 month postpartum	Too rare to be quantified, at least in highly resourced settings, where cases are in the setting of severe flares of immunologic diseases (SLE in primis). Still an issue in AKI; and in low resourced countries; not quantified in low-resourced countries, where it merges with dialysis need.
CKD progression	Decrease in GFR, rise in sCr, shift to a higher CKD stage	Differently assessed and estimated; may be linked to obstetric policy (anticipating delivery in the case of worsening of the kidney function); between 20% and 80% in advanced CKD. Probably not increased in early CKD stages.
Immunologic flares and neonatal SLE	Flares of immunologic diseases in pregnancy	Once thought to be increased in pregnancy, in particular in SLE, are probably a risk in patients who start pregnancy with an active disease, or with a recent flare-up. Definition of a “safe” zone is not uniformly agreed; in quiescent, well controlled diseases do not appear to be increased with respect to non-pregnant, carefully-matched controls.
Transplant rejection	Acute rejection in pregnancy	Similar to SLE, rejection episodes are not increased with respect to matched controls; may be an issue in unplanned pregnancies, in unstable patients.
Abortion	Fetal loss, before 21- 24 gestational weeks	May be increased in CKD, but data are scant. An issue in immunologic diseases (eventually, but not exclusively linked to the presence of LLAC) and in diabetic nephropathy.
Stillbirth	Delivery of a nonviable infant, after 21-24 gestational weeks	Probably not increased in early CKD, maybe an issue in dialysis patients; when not linked to extreme prematurity, may specifically linked to SLE, immunologic diseases and diabetic nephropathy.
Perinatal death	Death within 1 week – 1 month form delivery	Usually a result of extreme prematurity, which bears a risk of respiratory distress, neonatal sepsis, cerebral hemorrhage.
Small, very small baby	A baby weighting < 2500- 1500 g at birth	Has to be analyzed with respect to gestational age.
Preterm, early extremely preterm	Delivery before 37 – 34 or 28 completed gestational weeks	Increase in risk of preterm and early preterm delivery across CKD stages; extremely preterm may be an important issue in undiagnosed or late referred CKD and PE-AKI.
SGA (IUGR)	< 5th or <10th centile for gestational age	Strictly and inversely related to pre-term delivery; SGA and IUGR are probably related to risk for hypertension, metabolic syndrome and CKD in adulthood.
Malformations	Any kind of malformations	Malformations are not increased in CKD patients not treated by teratogen drugs (MMF, mTor inhibitors, ACEi, ARBS); exception: diabetic nephropathy (attributed to diabetes); hereditary diseases, such as PKD, reflux nephropathy, CAKUT may be evident at birth.
Hereditary kidney diseases	Any kind of CKD	Several forms of CKD recognize a hereditary pattern or predisposition; besides PKD, reflux and CAKUT, Alport’s disease, IgA, kidney tubular disorders and mitochondrial diseases have a genetic background, usually evident in adulthood and not always clearly elucidated.
CKD - hypertension	Higher risk of hypertension and CKD in adulthood	Late maturation of nephrons results in a lower nephron number in preterm babies; the risks are probably higher in SGA-IUGR babies than in pre-term babies adequate for gestational age.
Other long-term issues	Developmental disorders	Mainly due to prematurity, cerebral hemorrhage or neonatal sepsis, are not specific of CKD, but are a threat in all preterm babies.

*SLE* Systemic Lupus Erythematosus, *AKI* acute kidney injury, *GFR* glomerular filtration rate, *sCR* serum creatinine, *CKD* chronic kidney disease, *LLAC* Lupus-like anticoagulant, *PE-AKI* preeclampsia acute kidney injury, *SGA* small for gestational age, *IUGR* intrauterine growth restriction, *MMF* mycophenolate mofetil, *mTor* mechanistic target of rapamycin, *ACEi* angiotensin-converting-enzyme inhibitor, *ARBS* angiotensin II receptor blockers, *PKD* polycystic kidney disease, *CAKUT* congenital anomalies of the kidney and urinary tract, *IgA* immunoglobulin A

Hypertension and proteinuria at baseline are important modulators of pregnancy-related risks; among the risks, we know that malformations are not increased with respect to the overall population (out of the context of inherited diseases, such as reflux nephropathy, polycystic kidney disease, or congenital anomalies of the kidney and urinary tract), maternal death is unusual (in highly resourced countries), while the incidence of preterm delivery and of small for gestational age babies, intrinsically linked, is increased in stage 1 CKD patients, and rises with the worsening of kidney function. Likewise, the effect of pregnancy on CKD progression is not fully understood because of different study designs, obstetric policies, and duration of follow-up. Overall, short- and long- term decrease in kidney function is unusual in early CKD, but the risk increases as CKD severity increases [6, 7, 38–41, 44–48].

Pregnancy is a potential occasion for the initial diagnosis of CKD. In poorly or unevenly resourced countries, advanced CKD may be discovered only during pregnancy. The implications of dialysis initiation may present important clinical and ethical issues; in highly resourced countries with established prenatal care, the diagnosis of earlier stages of CKD may lead to more intensive therapy and surveillance [49–51].

#### Dialysis and transplantation

Fertility is reduced in ESRD; Australian and European data suggest a 1:10 ratio from general population to transplantation and from transplantation to dialysis (1:100 probability as compared to the general population) [52, 53]. The first sporadic cases of successful pregnancy on dialysis were described in the 70s, but in the new millennium this became an acknowledged real clinical possibility [8, 54, 55].

More than 1000 pregnancies have been reported in dialysis patients [55]. The most important advance has been the demonstration of a strong relationship between the intensity (frequency and duration) of the dialysis sessions and positive pregnancy results: thus, intensifying dialysis up to daily, is the current standard of care [8, 54]. Changing attitudes towards counselling women with advanced CKD may be impacted, with the knowledge of positive outcomes on dialysis for women and their offspring.

Fertility is partly restored after kidney transplantation [56–60]. However, even in an ideal situation (normal kidney function, no hypertension or proteinuria, at least 2 years after transplantation, without recent rejection episodes), the risk of complications is higher in women with transplanted kidneys than in the general population. However, if teratogen drugs are avoided (mycophenolic acid and rapamycine), the outcomes of pregnancy after kidney transplantation shares the same risk factors as CKD (kidney function, hypertension, and proteinuria) [59].

Experience with pregnancy in patients with a reduced renal function or failing kidney graft is limited and counseling is still forcedly based on personal experience or indirect evidence [61, 62]. Assisted fertilization techniques are increasingly popular in some settings, but dedicated studies in CKD patients are few; multiple pregnancies may bear an added risk in CKD patients, with both native and transplanted kidneys.

## Autoimmune diseases, women, and kidney disease

### What we know

Autoimmune diseases such as SLE, RA, and SS preferentially affect women and are characterized by systemic inflammation leading to target organ dysfunction, including kidneys. Sex differences in the incidence and severity of these diseases result from a complex interaction of hormonal, genetic, and epigenetic factors (Table 2). The public health burden of autoimmune diseases, which collectively represent a leading cause of morbidity and mortality among women throughout adulthood, is substantial [63–65].

**Table 2:** Sex differences in the incidence and severity of autoimmune diseases

		SLE	RA	SS
Peak Incidence		Reproductive age	Perimenopausal	After 50-60 years
Female/Male Ratio		Peak 15:1	Peak 4:1	Peak 14:1
		Total 9:1	After 60 years 1:1	Total 3:1
Influence of Estrogen	High levels	Negative	Positive	?
	Low levels	?	Negative	Negative

*SLE* Systemic Lupus Erythematosus, *RA* Rheumatoid Arthritis, *SS* Systemic Scleroderma

SLE is an autoimmune disease with multiple organ involvement, affecting approximately five million people worldwide; disproportionately predominant in women (9:1 female to male ratio) and individuals of non-European ancestry. The highest female predominance (up to 15:1) is in peak reproductive years. The biology of these differences has been explored: one explanation is the number of X chromosomes and genetic variants on the X chromosome [66–68]; another important etiological explanation is the role of estrogen in SLE. Estrogen’s primary effects are mediated by transcription activity of the intracellular estrogen receptors, whose profile is altered in T-cells from female SLE patients [69, 70]. Cathepsin S protein has recently been identified as a potential cause of lupus, triggering the immune system to attack healthy cells, particularly in females [71]. Numerous non-HLA genetic markers may predispose individuals of European, Hispanic, and Afro-American ancestry to lupus [72]. Susceptibility to SLE during pregnancy is also multifactorial; one factor being upregulation of IFN-α. Elevated IFN-α, expressed by the placenta, plays a pathogenic role in SLE, contributing both to the success of placental reproduction and to increased susceptibility to SLE [73]. Regulatory T-cells (which may be the key to cell modulating feto-maternal tolerance) have abnormalities of structure and function, and may contribute to pregnancy pathology in women with SLE and to challenges of managing them during pregnancy [74]. SLE affects kidneys in about 50% of patients, including glomerular, interstitial, and vascular lesions. Lupus nephritis is a major risk factor for overall morbidity and mortality in SLE, and despite potent therapies still leads to significant impairment of kidney function for many patients [75]. Kidney disease is a critical concern in counseling women with lupus considering pregnancy, with previous kidney involvement and lower C4 levels conferring high risk of active nephritis occurring in pregnancy [76]. Socioeconomic disparities are also linked to the health of patients with lupus. Poverty is associated with an increased long-term level of accumulated disease-associated damage and a 1.67-times increased likelihood of experiencing a clinically meaningful increase in damage. Frequency of adverse pregnancy outcomes in women with lupus is twofold higher in black and Hispanic women than in white women. In blacks, socioeconomic status was a determinant of pregnancy outcomes and a key contributor to adverse pregnancy outcomes [77, 78].

RA also preferentially affects women (4:1 ratio to men) with the peak incidence at age 45-55, coinciding with the perimenopausal years. This suggests a possible association between estrogen deficiency and disease onset. Female-to-male incidence ratio after age 60 years is approximately 1:1, potentially implicating changes in sex hormones in the development of RA, and a pattern of RA symptom improvement or even remission during pregnancy is well recognized [79–81]. Renal involvement in RA is relatively common and multifactorial and is a predictor of mortality in RA patients. The risk of CKD is significantly higher in patients with RA than in the general population. The development of CKD may result from several ongoing processes, including specific renal involvement associated with RA (e.g., glomerulonephritis, interstitial nephritis), chronic inflammation, comorbidities, and nephrotoxic anti-rheumatic drugs. The strong association between RA activity and AA amyloidosis increases morbidity and is the main cause of ESRD with RA and nephropathy. Importantly, some of the life-long and combined RA pharmacotherapy can lead to various renal side effects [82–84].

SS predominantly affects women (female-to-male ratios ranging from 3:1 to 14:1), with the peak incidence in the fifth and sixth decades. Estrogen may play a role in scleroderma pathogenesis through its stimulatory effect on transforming growth factor-beta 1 receptor and platelet-derived growth factor receptor [85]. Vasculopathy is an important disease-related manifestation in SS, and the low estrogenic state associated with menopause has been suggested to aggravate vascular manifestations in affected women [86]. SS can also be complicated by a number of different forms of kidney disease, including scleroderma renal crisis, which represents a form of malignant hypertension with acute renal failure; or more commonly ischemic nephropathy leading to slowly progressive CKD, accompanied by hypertension and albuminuria [78]. Normotensive acute renal failure in patients with SS may be caused by interstitial nephritis or ANCA vasculitis, a separate entity in scleroderma with poor outcome [87–89].

## Women, chronic kidney disease, and access to renal replacement therapies

### What we know

Although renal replacement therapy (RRT), including dialysis and transplantation is life-sustaining, not all patients receive RRT. The rate of ESRD treated by RRT differs greatly between countries and regions, and intricately depends on the economy of a country and health care system [90, 91]. Worldwide, only 50% of patients requiring RRT receive treatment [92], and in low and middle-income countries and regions, even less; in large parts of Sub-Saharan Africa, less than 2% of ESRD are treated by RRT [93]. The equality of access to RRT for women and girls is of particular concern because, in many societies, they are disadvantaged by discrimination rooted in sociocultural factors [94, 95].

## Sex differences in access to dialysis

At least 2.284 million people may have died prematurely due to lack of access to RRT with treatment gaps being much larger in low-income countries, with conservative estimates in Asia and Africa of 1.907 million and 432,000 people not receiving RRT. By 2030, the estimated number of RRT should be more than double to 5.439 million (3.899–7.640 million), with the most growth in Asia (0.968 million to a projected 2.162 million [1.71–3.14 million]) [92]. These numbers are derived from an extensive systematic review.

There are few data to compare the gender difference for the treatment gaps. Studies in Africa show that men were more likely to receive RRT than women [96, 97]. In Japan, the incidence of treated ESRD in females was less than half of that in males (3287 in males vs. 1764 women per million population treated) [91]: no explanations are given for this finding. One US study reports women having significantly higher odds ratio of 1.70 for late initiation of dialysis compared to men [98]. Awareness levels of previous kidney disease in women were reported much lower than in men (2.9% ± 1.6% in women vs. 17.9% ± 5.9% in men), which may contribute to later initiation of RRT [99].

Mortality rates are similar in men and women on dialysis, but the incident rates of some dialysis-associated complications and morbidity are higher in women. A US report of hospitalizations in 111,653 patients undergoing maintenance hemodialysis describes higher hospitalization rates in women, and higher risk for 30-day readmissions [100].

In addition, the prevalent use of arteriovenous fistula, which is associated with reduced mortality, complication, and costs, is lower among female than male hemodialysis patients [101]. This may be due to a number of different factors, including anatomical/surgical issues relating to vessel size, timing of referral, and attitudinal differences. This has not been systematically studied.

Dialysis dose, which is evaluated by Kt/V may result in under-dialysis in women who have an average smaller volume of urea distribution or total body water than men [102]. Women receiving dialysis have also been reported to have worse clinical parameters including anemia, nutrition, and quality of life [103]. Reasons are not certain.

## Sex differences in access to kidney transplantation

Transplantation represents the best form of RRT in patients without contraindications. Worldwide data describes that women are less likely than men to be kidney transplant recipients, either from a cadaveric or living donor, but are more likely to serve as living donors for kidney transplantation [104]. Data from different countries, including the US, France, China, and India, confirm differential kidney transplant rates (lower in women than men), less likelihood of women being registered on national transplant waiting lists, and longer time from dialysis initiation to listing. Mothers are more likely to be donors, as are female spouses [91, 105–108]. Sex inequality also exists in the pediatric population. A survey from 35 countries participating in the European Society for Pediatric Nephrology/European Renal Association-European Dialysis and Transplant Association Registry, reported girls had a lower access to renal transplantation than boys [109].

Socioeconomic factors undoubtedly play a role in the inequality of transplantation between sexes, especially in the low and middle-income countries and regions. Generally, men provide the major income for their family which may discourage them to donate kidneys. Different employment status and incomes between genders may contribute to sex differences in transplantation because employment and income status is usually associated with better healthcare insurance which cover the costs for transplantation. Psychosocial factors and education of women have been suggested as a contribution to sex disparity. US data found black women were less likely to want living donor kidney transplantation compared with men, despite being twice as likely as men to receive unsolicited offers for kidneys. They were also less likely to have been evaluated for a kidney transplant [110]. Other reports describe disparities in age and sex in access to kidney transplantation which originate at the time of pre-referral discussions about kidney transplantation; irrespective of age, women were more likely not to have had discussions with medical professionals. This result may imply that there is a need for better clinical guidelines and education for women, their social network, and their providers [111].

## Present and future: what we do not know

Given the data presented above with respect to pregnancy, AKI, autoimmune diseases, CKD, dialysis and transplantation, there are many unanswered questions. In high income countries with increasing maternal age and assisted fertilization, there may be an increase in PE which may impact future generations if associated with adverse fetal outcomes. The increase in in-vitro fertilization techniques for those of advanced maternal age may lead to multiple pregnancies, which may predispose to PE, intrauterine growth restriction, or both. Will this lead to an increase in CKD and CVD for women in the future?

Due to the high heterogeneity of CKD, we do not know if and how pregnancy outcomes are modulated by the different nephropathies, as besides the most common ones such as IgA or lupus nephropathy, diabetic nephropathy, and reflux nephropathy, evidence is scant [44, 45, 112–114]. How should we define preconception risks of pregnancy with respect to current proteinuria cut offs? Indications on when to start dialysis in pregnancy are not well established, nor is the specific role of frequency and duration. In those with kidney transplants, given the changing expanded donor policies, higher age at transplantation, and reduced fertility in older women, there may be changes in attitudes towards pregnancy with less than optimal kidney function [56, 60]. How this will impact short and long-term outcomes of mothers and their babies is not clear.

Teen pregnancies are very common in some parts of the world, and are often associated with low income and cultural levels. The uneven legal rules for assisted fertilization and the lack of systematic assessment of the kidney function point to the need for further research.

Despite elegant demonstrations for the role of sex hormones in vascular health and immunoregulation, the striking predominance in females of SLE, RA, and SS remains unexplained relative to other systemic diseases such as ANCA vasculitis and hemolytic-uremic syndrome. Note that thrombotic thrombocytopenic purpura has a higher incidence in women, though this is likely due to the association with other conditions more common in women. The incidence of kidney involvement in SLE during pregnancy and similarities/differences in those with PE have not been well studied. The role of different medications and responses to medications for autoimmune diseases relative to sex has also not been well studied.

More attention to similarities between conditions, the importance of sex hormones in inflammation, immune-modulation, and vascular health, may lead to important insights and clinical breakthroughs over time. If women are more likely to be living donors, at differential ages, does this impact both CVD risk, and risk for ESKD: have we studied this well enough, in the current era, with modern diagnostic criteria for CKD and sophisticated tools to understand renal reserve? Are the additional exposures that women have after living donation compounded by hormonal changes on vasculature as they age? And are the risks of CKD and PE increased in the younger female kidney living donor?

In the context of specific therapies for the treatment or delay of CKD progression, do we know if there are sex differences in therapeutic responses to ACEi/ARB? Should we look at dose finding/adjustments by sex? If vascular and immune biology is impacted by sex hormones as described earlier, do we know the impact of various therapies by level or ratio of sex hormones? In low-middle income countries how does changing economic and social cultures impact women’s health, and what is the nutritional impact on CKD of increasing predominance of obesity, diabetes, and hypertension?

## Summary

Women have unique risks for kidney diseases: kidney diseases, as well as issues related to access to care, have a profound impact on both the current and next generations. Advocating for improved access to care for women is critical to maintain the health of families, communities, and populations.

Focused studies on the unique contribution of sex hormones, or the interaction of sex hormones and other physiology, is important to improve our understanding of the progression of kidney diseases. Immunological conditions such as pregnancy (viewed as a state of tolerance to non-self) as well as SLE and other autoimmune and systemic conditions common in women, better studied may also lead to breakthroughs in understanding and care paradigms.

There is a clear need for higher awareness, timely diagnosis, and proper follow up of CKD in pregnancy. In turn, pregnancy may also be a valuable occasion for early diagnosis of CKD, allowing planning of therapeutic interventions.

On this occasion, World Kidney Day and the International Women’s Day 2018 are commemorated on the same day, offering us the opportunity to highlight the importance of women’s health and particularly their kidney health. On its 13th anniversary, World Kidney Day promotes affordable and equitable access to health education, healthcare, and prevention for all women and girls in the world.

The coinciding of World Kidney Day and International Women’s Day offers an opportunity to develop and define best practices and future research agendas, and ultimately, to optimize the outcomes of all people living with or at risk for kidney disease.

## References

[1] GBD 2015 Disease and Injury Incidence and Prevalence Collaborators (2016). Global, regional, and national incidence, prevalence, and years lived with disability for 310 diseases and injuries, 1990-2015: a systematic analysis for the Global Burden of Disease Study 2015. Lancet.

[2] von Dadelszen P, Payne B, Li J, Ansermino JM, Broughton Pipkin F, Côté A-M (2011). Prediction of adverse maternal outcomes in pre-eclampsia: development and validation of the fullPIERS model. Lancet.

[3] Mol BWJ, Roberts CT, Thangaratinam S, Magee LA, de Groot CJM, Hofmeyr GJ. Pre-eclampsia. Lancet 2016;387(10022):999–1011. 10.1016/S0140-6736(15)00070-7 26342729

[4] Vikse BE, Irgens LM, Leivestad T, Skjaerven R, Iversen BM (2008). Preeclampsia and the risk of end-stage renal disease. N Engl J Med.

[5] Theilen LH, Fraser A, Hollingshaus MS, Schliep KC, Varner MW, Smith KR (2016). All-Cause and Cause-Specific Mortality After Hypertensive Disease of Pregnancy. Obstet Gynecol.

[6] Piccoli GB, Cabiddu G, Attini R, Vigotti FN, Maxia S, Lepori N (2015). Risk of Adverse Pregnancy Outcomes in Women with CKD. J Am Soc Nephrol.

[7] Zhang J-J, Ma X-X, Hao L, Liu L-J, Lv J-C, Zhang H (2015). A Systematic Review and Meta-Analysis of Outcomes of Pregnancy in CKD and CKD Outcomes in Pregnancy. Clin J Am Soc Nephrol.

[8] Alkhunaizi A, Melamed N, Hladunewich MA (2015). Pregnancy in advanced chronic kidney disease and end-stage renal disease. Curr Opin Nephrol Hypertens.

[9] Piccoli GB, Cabiddu G, Castellino S, Gernone G, Santoro D, Moroni G (2017). A best practice position statement on the role of the nephrologist in the prevention and follow-up of preeclampsia: the Italian study group on kidney and pregnancy. J Nephrol..

[10] Liu Y, Ma X, Zheng J, Liu X, Yan T (2017). Pregnancy outcomes in patients with acute kidney injury during pregnancy: a systematic review and meta-analysis. BMC Pregnancy Childbirth.

[11] Jim B, Garovic VD (2017). Acute Kidney Injury in Pregnancy. Semin Nephrol.

[12] Acharya A (2016). Management of Acute Kidney Injury in Pregnancy for the Obstetrician. Obstet Gynecol Clin N Am.

[13] Iseki K (2008). Gender differences in chronic kidney disease. Kidney Int.

[14] Nitsch D, Grams M, Sang Y, Black C, Cirillo M, Djurdjev O (2013). Associations of estimated glomerular filtration rate and albuminuria with mortality and renal failure by sex: a meta-analysis. BMJ.

[15] Levin A, Djurdjev O, Beaulieu M, Er L (2008). Variability and risk factors for kidney disease progression and death following attainment of stage 4 CKD in a referred cohort. Am J Kidney Dis.

[16] Weiner DE, Tighiouart H, Elsayed EF, Griffith JL, Salem DN, Levey AS (2007). The Framingham predictive instrument in chronic kidney disease. J Am Coll Cardiol.

[17] Oladapo OT, Adetoro OO, Ekele BA, Chama C, Etuk SJ, Aboyeji AP (2016). When getting there is not enough: a nationwide cross-sectional study of 998 maternal deaths and 1451 near-misses in public tertiary hospitals in a low-income country. BJOG.

[18] Tranquilli AL, Dekker G, Magee L, Roberts J, Sibai BM, Steyn W (2014). The classification, diagnosis and management of the hypertensive disorders of pregnancy: A revised statement from the ISSHP. Pregnancy Hypertens.

[19] Liu Y, Bao H, Jiang Z, Huang Y, Wang N (2015). Pregnancy-related Acute Kidney Injury and a Review of the Literature in China. Intern Med.

[20] Prakash J, Pant P, Prakash S, Sivasankar M, Vohra R, Doley PK (2016). Changing picture of acute kidney injury in pregnancy: Study of 259 cases over a period of 33 years. Indian J Nephrol.

[21] Ibarra-Hernández M, Orozco-Guillén OA, de la Alcantar-Vallín ML, et al. Acute kidney injury in pregnancy and the role of underlying CKD: a point of view fromMéxico. JNephrol. 2017;30:773–80. 10.1007/s40620-017-0444-4 29022223

[22] Blázquez A, García D, Rodríguez A, Vassena R, Figueras F, Vernaeve V (2016). Is oocyte donation a risk factor for preeclampsia? A systematic review and meta-analysis. J Assist Reprod Genet.

[23] O’Gorman N, Wright D, Poon LC, Rolnik DL, Syngelaki A, de Alvarado M (2017). Multicenter screening for pre-eclampsia by maternal factors and biomarkers at 11-13 weeks’ gestation: comparison with NICE guidelines and ACOG recommendations. Ultrasound Obstet Gynecol.

[24] Zeisler H, Llurba E, Chantraine F, Vatish M, Sennström M, Staff AC (2016). Predictive Value of the sFlt-1:PlGF Ratio in Women with Suspected Preeclampsia. N Engl J Med.

[25] Garovic VD (2014). The Role of the Podocyte in Preeclampsia. Clin J Am Soc Nephrol.

[26] Wide-Swensson D, Strevens H, Willner J (2007). Antepartum percutaneous renal biopsy. Int J Gynaecol Obstet.

[27] Shiiki H, Dohi K, Hanatani M, Fujii Y, Sanai H, Ichijo M (1990). Focal and segmental glomerulosclerosis in preeclamptic patients with nephrotic syndrome. Am J Nephrol.

[28] Linsell L, Malouf R, Morris J, Kurinczuk JJ, Marlow N (2017). Risk Factor Models for Neurodevelopmental Outcomes in Children Born Very Preterm or With Very Low Birth Weight: A Systematic Review of Methodology and Reporting. Am J Epidemiol.

[29] Guellec I, Lapillonne A, Marret S, Picaud J-C, Mitanchez D, Charkaluk M-L (2016). Effect of Intra- and Extrauterine Growth on Long-Term Neurologic Outcomes of Very Preterm Infants. J Pediatr.

[30] Moore T, Hennessy EM, Myles J, Johnson SJ, Draper ES, Costeloe KL (2012). Neurological and developmental outcome in extremely preterm children born in England in 1995 and 2006: the EPICure studies. BMJ.

[31] Guillén Ú, DeMauro S, Ma L, Zupancic J, Roberts R, Schmidt B (2012). Relationship Between Attrition and Neurodevelopmental Impairment Rates in Extremely Preterm Infants at 18 to 24 Months: A Systematic Review. Arch Pediatr Adolesc Med.

[32] Ranke MB, Schweizer R, Rodemann SM, Bevot A, Martin DD, Goelz R (2015). Schoolchildren born VLBW or VLGA show height-related changes in body composition and muscle function but no evidence of metabolic syndrome risk factors. Results from the NEOLONG study. J Pediatr Endocrinol Metab.

[33] Castanys-Muñoz E, Kennedy K, Castañeda-Gutiérrez E, Forsyth S, Godfrey KM, Koletzko B (2017). Systematic review indicates postnatal growth in term infants born small-for-gestational-age being associated with later neurocognitive and metabolic outcomes. Acta Paediatr.

[34] Ong KK, Kennedy K, Castañeda-Gutiérrez E, Forsyth S, Godfrey KM, Koletzko B (2015). Postnatal growth in preterm infants and later health outcomes: a systematic review. Acta Paediatr.

[35] Low Birth Weight and Nephron Number Working Group (2017). The Impact of Kidney Development on the Life Course: A Consensus Document for Action. Nephron.

[36] Luyckx VA, Bertram JF, Brenner BM, Fall C, Hoy WE, Ozanne SE (2013). Effect of fetal and child health on kidney development and long-term risk of hypertension and kidney disease. Lancet.

[37] Luyckx VA, Brenner BM (2015). Birth weight, malnutrition and kidney-associated outcomes--a global concern. Nat Rev Nephrol..

[38] Davison JM, Lindheimer MD (2011). Pregnancy and chronic kidney disease. Semin Nephrol.

[39] Hall M (2016). Pregnancy in Women With CKD: A Success Story. Am J Kidney Dis.

[40] Nevis IF, Reitsma A, Dominic A, McDonald S, Thabane L, Akl EA (2011). Pregnancy outcomes in women with chronic kidney disease: a systematic review. Clin J Am Soc Nephrol.

[41] Cabiddu G, Castellino S, Gernone G, Santoro D, Moroni G, Giannattasio M (2016). A best practice position statement on pregnancy in chronic kidney disease: the Italian Study Group on Kidney and Pregnancy. J Nephrol.

[42] Garg AX, Nevis IF, McArthur E, Sontrop JM, Koval JJ, Lam NN (2015). Gestational hypertension and preeclampsia in living kidney donors. N Engl J Med.

[43] Josephson MA (2009). Transplantation: pregnancy after kidney donation: more questions than answers. Nat Rev Nephrol.

[44] Gianfreda D, Quaglini S, Frontini G, Raffiotta F, Messa P, Moroni G (2017). Does pregnancy have any impact on long term damage accrual and on the outcome of lupus nephritis?. J Autoimmun.

[45] Blom K, Odutayo A, Bramham K, Hladunewich MA. Pregnancy and glomerular disease: a systematic review of the literature with management guidelines. Clin J Am Soc Nephrol. 2017;12:1862–1872. 10.2215/CJN.00130117 PMC5672957 28522651

[46] Imbasciati E, Gregorini G, Cabiddu G, Gammaro L, Ambroso G, Del Giudice A (2007). Pregnancy in CKD stages 3 to 5: fetal and maternal outcomes. Am J Kidney Dis.

[47] Fischer MJ (2007). Chronic kidney disease and pregnancy: maternal and fetal outcomes. Adv Chronic Kidney Dis.

[48] Bramham K (2017). Diabetic Nephropathy and Pregnancy. Semin Nephrol.

[49] Eswarappa M, Rakesh M, Sonika P, Snigdha K, Midhun M, Kaushik K (2017). Spectrum of renal injury in pregnancy-induced hypertension: Experience from a single center in India. Saudi Journal of Kidney Diseases and Transplantation.

[50] Prakash J (2012). The kidney in pregnancy: A journey of three decades. Indian J Nephrol..

[51] Piccoli GB, Fassio F, Attini R, Parisi S, Biolcati M, Ferraresi M (2012). Pregnancy in CKD: whom should we follow and why?. Nephrol Dial Transplant.

[52] Piccoli GB, Cabiddu G, Daidone G, Guzzo G, Maxia S, Ciniglio I (2014). The children of dialysis: live-born babies from on-dialysis mothers in Italy--an epidemiological perspective comparing dialysis, kidney transplantation and the overall population. Nephrol Dial Transplant.

[53] Jesudason S, Grace BS, McDonald SP (2014). Pregnancy Outcomes According to Dialysis Commencing Before or After Conception in Women with ESRD. Clin J Am Soc Nephrol.

[54] Hladunewich MA, Hou S, Odutayo A, Cornelis T, Pierratos A, Goldstein M (2014). Intensive hemodialysis associates with improved pregnancy outcomes: a Canadian and United States cohort comparison. J Am Soc Nephrol.

[55] Piccoli GB, Minelli F, Versino E, Cabiddu G, Attini R, Vigotti FN (2016). Pregnancy in dialysis patients in the new millennium: a systematic review and meta-regression analysis correlating dialysis schedules and pregnancy outcomes. Nephrol Dial Transplant.

[56] Deshpande NA, James NT, Kucirka LM, Boyarsky BJ, Garonzik-Wang JM, Montgomery RA (2011). Pregnancy outcomes in kidney transplant recipients: a systematic review and meta-analysis. Am J Transplant.

[57] Deshpande NA, Coscia LA, Gomez-Lobo V, Moritz MJ, Armenti VT (2013). Pregnancy after solid organ transplantation: a guide for obstetric management. Rev Obstet Gynecol.

[58] Bramham K, Nelson-Piercy C, Gao H, Pierce M, Bush N, Spark P (2013). Pregnancy in renal transplant recipients: a UK national cohort study. Clin J Am Soc Nephrol.

[59] Piccoli GB, Cabiddu G, Attini R, Gerbino M, Todeschini P, Perrino ML (2017). Outcomes of Pregnancies After Kidney Transplantation: Lessons Learned from CKD. A Comparison of Transplanted, Nontransplanted Chronic Kidney Disease Patients and Low-Risk Pregnancies: A Multicenter Nationwide Analysis. Transplantation.

[60] Webster P, Lightstone L, McKay DB, Josephson MA (2017). Pregnancy in chronic kidney disease and kidney transplantation. Kidney Int.

[61] Pietrzak B, Mazanowska N, Kociszewska-Najman B, Szymusik I, Grzechocińska B, Pazik J (2015). Successful Pregnancy Outcome after In Vitro Fertilization in a Kidney Graft Recipient: A Case Report and Literature Review. Ann Transplant.

[62] Norrman E, Bergh C, Wennerholm U-B (2015). Pregnancy outcome and long-term follow-up after in vitro fertilization in women with renal transplantation. Hum Reprod.

[63] Tedeschi SK, Bermas B, Costenbader KH (2013). Sexual disparities in the incidence and course of SLE and RA. Clin Immunol.

[64] Marder W, Vinet É, Somers EC. Rheumatic autoimmune diseases in women and midlife health. Womens Midlife Health [Internet]. 2015 [cited 2017 Oct 19];1. Available from: https://www.ncbi.nlm.nih.gov/pmc/articles/PMC5444314/. 10.1186/s40695-015-0012-9 PMC5444314 28553545

[65] Ortona E, Pierdominici M, Maselli A, Veroni C, Aloisi F, Shoenfeld Y (2016). Sex-based differences in autoimmune diseases. Ann Ist Super Sanita.

[66] Petri M (2002). Epidemiology of systemic lupus erythematosus. Best Pract Res Clin Rheumatol.

[67] Weckerle CE, Niewold TB (2011). The unexplained female predominance of systemic lupus erythematosus: clues from genetic and cytokine studies. Clin Rev Allergy Immunol.

[68] Scofield RH, Bruner GR, Namjou B, Kimberly RP, Ramsey-Goldman R, Petri M (2008). Klinefelter’s syndrome (47, XXY) in male systemic lupus erythematosus patients: support for the notion of a gene-dose effect from the X chromosome. Arthritis Rheum.

[69] Pierdominici M, Ortona E (2013). Estrogen Impact on Autoimmunity Onset and Progression: the Paradigm of Systemic Lupus Erythematosus. Int Trends in Immun.

[70] Maselli A, Conti F, Alessandri C, Colasanti T, Barbati C, Vomero M, et al. Low expression of estrogen receptor β in T lymphocytes and high serum levels of anti-estrogen receptor α antibodies impact disease activity in female patients with systemic lupus erythematosus. Biol Sex Differ [Internet]. 2016 Jan 12 [cited 2017 Oct 19];7. Available from: https://www.ncbi.nlm.nih.gov/pmc/articles/PMC4709986/. 10.1186/s13293-016-0057-y PMC4709986 26759713

[71] Kim SJ, Schätzle S, Ahmed SS, Haap W, Jang SH, Gregersen PK (2017). Increased cathepsin S in Prdm1(−/−) dendritic cells alters the TFH cell repertoire and contributes to lupus. Nat Immunol.

[72] Langefeld CD, Ainsworth HC, Cunninghame Graham DS, Kelly JA, Comeau ME, Marion MC (2017). Transancestral mapping and genetic load in systemic lupus erythematosus. Nat Commun.

[73] Niewold TB, Hua J, Lehman TJA, Harley JB, Crow MK (2007). High serum IFN-alpha activity is a heritable risk factor for systemic lupus erythematosus. Genes Immun.

[74] Tower C, Mathen S, Crocker I, Bruce IN (2013). Regulatory T cells in Systemic Lupus Erythematosus and Pregnancy. Am J Reprod Immunol.

[75] Almaani S, Meara A, Rovin BH (2016). Update on Lupus Nephritis. CJASN.

[76] Buyon JP, Kim MY, Guerra MM, Lu S, Reeves E, Petri M (2017). Kidney Outcomes and Risk Factors for Nephritis (Flare/De Novo) in a Multiethnic Cohort of Pregnant Patients with Lupus. Clin J Am Soc Nephrol.

[77] Yelin E, Yazdany J, Trupin L (2017). Relationship Between Process of Care and a Subsequent Increase in Damage in Systemic Lupus Erythematosus. Arthritis Care & Research.

[78] Kaplowitz ET, Ferguson S, Guerra M, et al. Socioeconomic status contributes to racial/ethnic disparities in adverse pregnancy outcomes among women with systemic lupus erythematosus. Arthritis Care Res [Hoboken]. 2017. 10.1002/acr.23263 28480528

[79] Myasoedova E, Crowson CS, Kremers HM, Therneau TM, Gabriel SE (2010). Is the incidence of rheumatoid arthritis rising?: results from Olmsted County, Minnesota, 1955-2007. Arthritis Rheum.

[80] Goemaere S, Ackerman C, Goethals K, De Keyser F, Van der Straeten C, Verbruggen G (1990). Onset of symptoms of rheumatoid arthritis in relation to age, sex and menopausal transition. J Rheumatol.

[81] de Man YA, Dolhain RJEM, van de Geijn FE, Willemsen SP, Hazes JMW (2008). Disease activity of rheumatoid arthritis during pregnancy: results from a nationwide prospective study. Arthritis Rheum.

[82] Icardi A, Araghi P, Ciabattoni M, Romano U, Lazzarini P, Bianchi G (2003). Kidney involvement in rheumatoid arthritis. Reumatismo.

[83] Anders H-J, Vielhauer V (2011). Renal co-morbidity in patients with rheumatic diseases. Arthritis Research & Therapy.

[84] Chiu H-Y, Huang H-L, Li C-H, Chen H-A, Yeh C-L, Chiu S-H, et al. Increased Risk of Chronic Kidney Disease in Rheumatoid Arthritis Associated with Cardiovascular Complications – A National Population-Based Cohort Study. PLoS One 2015;10(9):e0136508. 10.1371/journal.pone.0136508 PMC4583248 26406879

[85] Vinet É, Bernatsky S, Hudson M, Pineau CA, Baron M (2014). Effect of menopause on the modified Rodnan skin score in systemic sclerosis. Arthritis Research & Therapy.

[86] Sammaritano LR (2012). Menopause in patients with autoimmune diseases. Autoimmun Rev.

[87] Penn H, Denton CP (2008). Diagnosis, management and prevention of scleroderma renal disease. Curr Opin Rheumatol.

[88] Anders HJ, Wiebecke B, Haedecke C, Sanden S, Combe C, Schlöndorff D (1999). MPO-ANCA-Positive crescentic glomerulonephritis: a distinct entity of scleroderma renal disease?. Am J Kidney Dis.

[89] Zakharova EV, Makarova TA, Stolyarevich ES. ANCA-Associated Vasculitis in Patient with CREST-Syndrome - Case Report [Internet]. [cited 2017 Oct 19]. Available from: https://www.peertechz.com/Clinical-Nephrology/ACN-2-115.php.

[90] Eckardt K-U, Coresh J, Devuyst O, Johnson RJ, Köttgen A, Levey AS (2013). Evolving importance of kidney disease: from subspecialty to global health burden. Lancet.

[91] Saran R, Robinson B, Abbott KC, Agodoa LYC, Albertus P, Ayanian J (2017). US Renal Data System 2016 Annual Data Report: Epidemiology of Kidney Disease in the United States. Am J Kidney Dis.

[92] Liyanage T, Ninomiya T, Jha V, Neal B, Patrice HM, Okpechi I (2015). Worldwide access to treatment for end-stage kidney disease: a systematic review. Lancet.

[93] Ojo A (2014). Addressing the global burden of chronic kidney disease through clinical and translational research. Trans Am Clin Climatol Assoc.

[94] WHO. Addressing gender within primary health care reforms. In: WHO editor. Gender, women and primary health care renewal: a discussion paper [Internet]. [cited 2017 4Oct 19]. Available from: http://apps.who.int/iris/bitstream/10665/44430/1/9789241564038_eng.pdf.

[95] Eguavoen ANT, Odiagbe SO, Obetoh GI (2007). The Status of Women, Sex Preference, Decision-Making and Fertility Control in Ekpoma Community of Nigeria. J Soc Sci.

[96] Halle MP, Takongue C, Kengne AP, Kaze FF, Ngu KB (2015). Epidemiological profile of patients with end stage renal disease in a referral hospital in Cameroon. BMC Nephrol.

[97] Ajayi S, Raji Y, Bello T, Jinadu L, Salako B (2016). Unaffordability of renal replacement therapy in Nigeria. Hong Kong Journal of Nephrology.

[98] Kausz AT, Obrador GT, Arora P, Ruthazer R, Levey AS, Pereira BJ (2000). Late initiation of dialysis among women and ethnic minorities in the United States. J Am Soc Nephrol.

[99] Coresh J, Byrd-Holt D, Astor BC, Briggs JP, Eggers PW, Lacher DA (2005). Chronic kidney disease awareness, prevalence, and trends among U.S. adults, 1999 to 2000. J Am Soc Nephrol.

[100] Adams SV, Rivara M, Streja E, Cheung AK, Arah OA, Kalantar-Zadeh K (2017). Sex Differences in Hospitalizations with Maintenance Hemodialysis. J Am Soc Nephrol.

[101] Ethier J, Mendelssohn DC, Elder SJ, Hasegawa T, Akizawa T, Akiba T (2008). Vascular access use and outcomes: an international perspective from the Dialysis Outcomes and Practice Patterns Study. Nephrol Dial Transplant.

[102] Depner TA (2003). Prescribing Hemodialysis: The Role of Gender. Adv Ren Replace Ther.

[103] Sehgal AR (2000). Outcomes of renal replacement therapy among blacks and women. Am J Kidney Dis.

[104] Jindal RM, Ryan JJ, Sajjad I, Murthy MH, Baines LS (2005). Kidney transplantation and gender disparity. Am J Nephrol.

[105] Couchoud C, Bayat S, Villar E, Jacquelinet C, Ecochard R (2012). REIN registry. A new approach for measuring gender disparity in access to renal transplantation waiting lists. Transplantation.

[106] Liu G, Li X, Liu T, Zhao X, Zhang S, Wang J (2013). Gender disparity of living donor renal transplantation in East China. Clin Transpl.

[107] Naghibi O, Naghibi M, Nazemian F (2008). Gender disparity in kidney transplantation. Saudi J Kidney Dis Transpl.

[108] Bal MM, Saikia B (2007). Gender bias in renal transplantation: are women alone donating kidneys in India?. Transplant Proc.

[109] Hogan J, Couchoud C, Bonthuis M, Groothoff JW, Jager KJ, Schaefer F (2016). Gender Disparities in Access to Pediatric Renal Transplantation in Europe: Data from the ESPN/ERA-EDTA Registry. Am J Transplant.

[110] Gillespie A, Hammer H, Kolenikov S, Polychronopoulou A, Ouzienko V, Obradovic Z (2014). Sex Differences and Attitudes toward Living Donor Kidney Transplantation among Urban Black Patients on Hemodialysis. Clin J Am Soc Nephrol.

[111] Salter ML, McAdams-Demarco MA, Law A, Kamil RJ, Meoni LA, Jaar BG (2014). Age and sex disparities in discussions about kidney transplantation in adults undergoing dialysis. J Am Geriatr Soc.

[112] Piccoli GB, Attini R, Cabiddu G, Kooij I, Fassio F, Gerbino M (2017). Maternal-foetal outcomes in pregnant women with glomerulonephritides. Are all glomerulonephritides alike in pregnancy?. J Autoimmun.

[113] Seeger H, Salfeld P, Eisel R, Wagner CA, Mohebbi N (2017). Complicated pregnancies in inherited distal renal tubular acidosis: importance of acid-base balance. J Nephrol..

[114] Yefet E, Tovbin D, Nachum Z (2016). Pregnancy outcomes in patients with Alport syndrome. Arch Gynecol Obstet.

